# Gene expression changes in therapeutic ultrasound-treated venous leg ulcers

**DOI:** 10.3389/fmed.2023.1144182

**Published:** 2023-03-30

**Authors:** Olivia Boerman, Zahidur Abedin, Rose Ann DiMaria-Ghalili, Michael S. Weingarten, Michael Neidrauer, Peter A. Lewin, Kara L. Spiller

**Affiliations:** ^1^School of Biomedical Engineering, Science and Health Systems, Drexel University, Philadelphia, PA, United States; ^2^Biomedical Engineering, Bucknell University, Lewisburg, PA, United States; ^3^Division of Molecular Biology - Research Services, PrimBio Research Institute, Exton, PA, United States; ^4^Department of Nursing, College of Nursing and Health Professions, Drexel University, Philadelphia, PA, United States; ^5^Department of Surgery, College of Medicine, Drexel University, Philadelphia, PA, United States

**Keywords:** venous leg ulcer, ultrasound, gene expression, ampliseq, translational medicine

## Abstract

**Introduction:**

Low-frequency, low-intensity ultrasound has been previously shown to promote healing of chronic wounds in humans, but mechanisms behind these effects are poorly understood. The purpose of this study was to evaluate gene expression differences in debrided human venous ulcer tissue from patients treated with low-frequency (20 kHz), low-intensity (100 mW/cm^2^) ultrasound compared to a sham treatment in an effort to better understand the potential biological mechanisms.

**Methods:**

Debrided venous ulcer tissue was collected from 32 subjects one week after sham treatment or low-frequency, low-intensity ultrasound treatment. Of these samples, 7 samples (3 ultrasound treated and 4 sham treated) yielded sufficient quality total RNA for analysis by ultra-high multiplexed PCR (Ampliseq) and expression of more than 24,000 genes was analyzed. 477 genes were found to be significantly differentially expressed between the ultrasound and sham groups using cut-off values of *p* < 0.05 and fold change of 2.

**Results and Discussion:**

The top differentially expressed genes included those involved in regulation of cell metabolism, proliferation, and immune cell signaling. Gene set enrichment analysis identified 20 significantly enriched gene sets from upregulated genes and 4 significantly enriched gene sets from downregulated genes. Most of the enriched gene sets from upregulated genes were related to cell-cell signaling pathways. The most significantly enriched gene set from downregulated genes was the inflammatory response gene set. These findings show that therapeutic ultrasound influences cellular behavior in chronic wounds as early as 1 week after application. Considering the well-known role of chronic inflammation in impairing wound healing in chronic wounds, these results suggest that a downregulation of inflammatory genes is a possible biological mechanism of ultrasound-mediated venous chronic wound healing. Such increased understanding may ultimately lead to the enhancement of ultrasound devices to accelerate chronic wound healing and increase patient quality of life.

## Introduction

Chronic venous leg ulcers (VLUs), defined as wounds that do not heal in 4–6 weeks, are a significant health care burden and affect 1–2% of the worldwide adult population with increased incidence in women and older adults (1–3). Because of their slow healing time, VLUs cause substantial socioeconomic impact, costing up to $2 billion per year in the United States alone (4, 5). The current standard of care for chronic VLUs includes weekly wound dressing changes, the use of compression bandages, and sharp tissue debridement (5). Although 93% of VLUs will heal in 12 months (6), the recurrence rate within 3 months is 70% (6). There is a need for more effective chronic wound therapies.

One promising therapeutic approach is the application of low-frequency, low-intensity ultrasound (LFLI US), which has been shown in clinical trials to significantly accelerate wound healing of chronic VLUs (7–10). In a double-blind study, patients with VLUs were treated with a non-operational sham device or with LFLI US operating at 20 kHz and 100 mW/cm^2^ once a week for 15 min or 45 min for 8 weeks (8). Patients in the 15-min treatment group experienced full wound closure after 4 weeks (*n* = 5), while patients in the sham group saw an increase in wound size (*n* = 5), and patients in the 45-min treatment group did not see full wound closure in the duration of the study (*n* = 5) (8). Despite these promising clinical results, the optimal operating parameters of therapeutic ultrasound are still unknown, and the mechanisms are poorly understood. Furthermore, these mechanisms depend on ultrasound exposure parameters, such as frequency, spatial distribution of the pressure amplitude or intensity, and time duration. A better understanding of the mechanisms of action by which therapeutic ultrasound promotes wound closure could lead to optimization of ultrasound device parameters and increase the rate of wound healing.

Some studies have explored the potential biological mechanism of US in animal models (9, 11). For example, Lyu et al. demonstrated that US stimulation (operating at 2 MHz) increased wound healing in a diabetic rat model, and suggested that the effect might be *via* the Rac1 mechanotransduction pathway, which was also increased (9). Moreover, Maan et al. showed that the delivery of low frequency (40 kHz) US through a saline mist increased wound healing in diabetic mice, and suggested that stimulation of angiogenesis might be a potential mechanism (11). These potential mechanisms also have been put forth as mediators of US-stimulated bone fracture healing, an indication in which LFLI US has been more thoroughly evaluated compared to chronic wounds (12, 13). However, wound healing in animals, particularly rodents, is fundamentally different than wound healing in humans as it primarily occurs *via* contraction (14, 15) and because of differences in the inflammatory response to injury (16). Therefore, there is a need to investigate mechanisms of wound healing in humans.

The goal of this study was to explore the possible hypotheses for the biological mechanisms of therapeutic ultrasound-assisted wound healing in human patients by analyzing gene expression in tissue collected from VLUs. Expression of a panel of inflammation-related genes has been previously linked to healing in ultrasound-treated human diabetic foot ulcers using tissue collected during routine wound debridement (17). Therefore, in this study, we similarly analyzed debrided wound tissue and assessed the whole transcriptome to identify possible mechanisms. We collected debrided human chronic wound tissue 1 week after treatment as part of a double-blinded study in which patients were treated with ultrasound operating at 20 kHz and 100 mW/cm^2^ (SPTP) or a nonoperational sham device. This tissue was processed using an ultra-high multiplexed PCR (Ampliseq), which was selected because of its favorable profile in detecting transcriptional changes in even low-input samples (18).

## Materials and methods

### Study design

As part of an ongoing double-blind human clinical study investigating the efficacy of therapeutic ultrasound for chronic wound healing (ClinicalTrials.gov Identifier: NCT03041844), 48 patients with chronic VLUs were recruited from Drexel University Comprehensive Wound Healing Program. The study protocol was reviewed and approved by the Drexel University Institutional Review Board (IRB).

Inclusion criteria for participants to enroll into the study were as follows: have a VLU that has been documented for at least 8 weeks without complete re-epithelialization, a VLU size of larger than 0.75 cm^2^, VLU must be present on the lower extremities non-weight-bearing areas. The exclusion criteria included: VLU is secondary to any connective tissue disorder or blood dyscrasias, severe vascular insufficiency (ankle-brachial index lower than 0.75 or toe-brachial index below 0.5), active and untreated infection, acute deep venous thrombosis, cutaneous malignancy present on involved extremity, active (or past 6 months) cancer treatment, presence of both a diabetic ulcer and venous ulcer on the same extremity, known allergy to Tegaderm (a polyurethane dressing), pregnancy, individuals younger than 18 years of age, prisoners, individuals not able to read or speak English, Spanish, or Mandarin, and adults unable to consent. Finally, subjects with concomitant arterial disease were excluded by the presence of a palpable pedal pulse in the extremity on physical examination or a toe/bracial index of 0.6 or greater. Subjects were asked for a list of their comorbidities or the subject’s list of comorbidities were extracted from the physician’s medical record. Subject demographics are shown in Table 1.

**Table 1:** Subject demographics of analyzed wound tissue samples.

Variable	Ultrasound treated (*n* = 3)			Sham treated (*n* = 4)			
Healed during study?	No	No	No	No	No	Yes; healed at week 8	Yes; healed at week 15
Baseline wound surface area (cm^2^)	4.5	31.5	60	36	54.18	25.2	1.36
Wound surface area after 1 week (at time of sample collection) (cm^2^)	3.75	29.4	78	57	43.68	27.06	1.44
Difference in wound surface area from baseline to week 1 (cm^2^)	−0.75	−2.1	+18	+21	−10.5	+1.86	+0.08
Sex	Female	Male	Male	Male	Male	Female	Female
Age	58	45	49	56	30	47	66
Race	Black or African American	Black or African American	Black or African American	Black or African American	White	Black or African American	White
Ethnicity	Not Hispanic or Latino	Not Hispanic or Latino	Not Hispanic or Latino	Not Hispanic or Latino	Not Hispanic or Latino	Not Hispanic or Latino	Not Hispanic or Latino
BMI	35.99	24.73	62.62	34.66	25.38	40.09	39.31
Statin Use	No	No	Yes	No	No	Yes	No
Diabetes	Pre-diabetes	No	No	Yes	No	Pre-diabetes	No
Kidney Failure	Yes	No	No	Yes	No	No	No
Hypertension	No	No	Yes	No	No	No	Yes
Heart Disease	Yes	No	Yes	Yes	Yes	No	No
Education	>High School	High School	<High School	>High School	>High School	>High School	High School
Employment Status	Receives Disability	Receives Disability	Receives Disability	Work outside the home	Work outside the home	Other	Retired

Weekly study visits took place in an outpatient wound clinic. Subjects randomized to the therapeutic ultrasound group were topically treated with 20 kHz, 100 mW/cm^2^ for 15 min while patients in the sham group were treated with a non-operating device for 15 min. The ultrasound devices used in this study have been previously described in detail in Ngo et al. (10). After completion of the ultrasound or sham treatment, the vascular surgeon performed tissue debridement using a surgical scalpel and participants received standard wound care. All tissue samples analyzed in this study were collected 1 week following ultrasound or sham treatment. Subjects who did not return to the clinic 1 week following ultrasound or sham treatment were considered lost to follow up.

**Figure 1:**
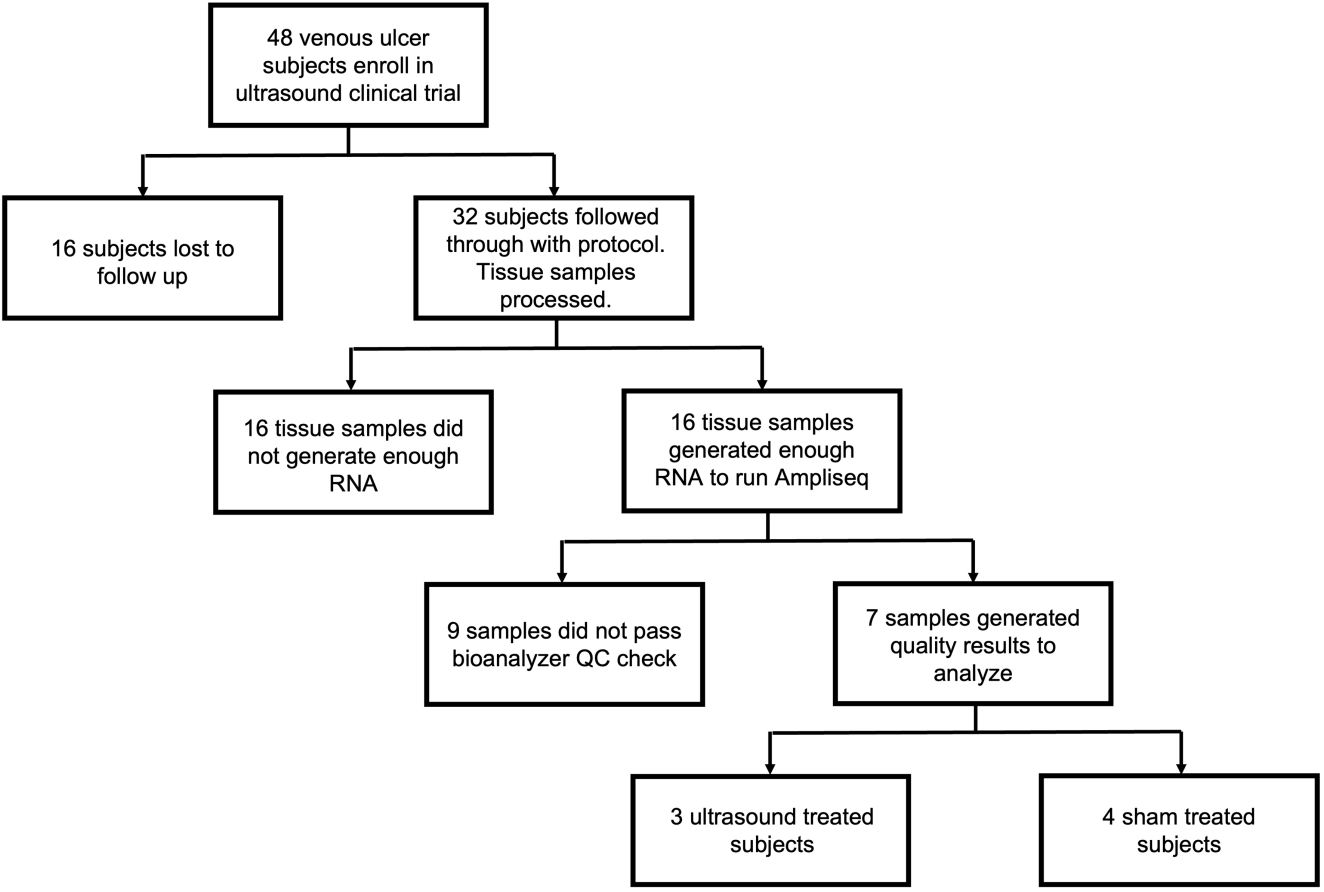
Flow chart outlining subject enrollment, sample collection, quality control, and final gene expression analysis for ultrasound-treated subjects (*n* = 3) and sham-treated subjects (*n* = 4).

Of the 48 subjects enrolled in the study, 16 subjects were lost to follow up resulting in 32 tissue samples processed (Figure 1). Tissue samples were processed using the methods described in subsequent sections. Of the 32 processed samples, 16 did not yield RNA of sufficient quantity, and of those, 9 did not yield RNA of sufficient quality for library preparation. Thus, 7 tissue samples were ultimately analyzed, and these comprised 3 from the ultrasound treatment group and 4 from the sham group. Healing outcome was determined by considering complete wound closure at week 16 or earlier as healed and incomplete wound closure at that time point as non-healed.

### Debrided tissue collection

All participants underwent VLU debridement by a vascular surgeon as part of standard wound care regimen during each visit. Sharp debridement was conducted using a scalpel or curette after removing the overlying biofilm and necrotic tissue. The entirety of the debrided tissue was collected, as opposed to only wound bed or edge, because separate debridement of different wound regions is not routinely conducted in clinical practice, although it is noted that there are biological differences between the wound regions (19, 20). For similar reasons, intact skin tissue was not collected. Finally, wound histology was not conducted so that sample collection methods would not deviate from the normal standard of care debridement procedures. In addition, all of the debrided tissue was required to isolate sufficient quantity and quality of RNA for gene expression profiling. Debrided tissue was immediately stored in RNAlater® Solution (Ambion, Austin, TX, United States) at 4°C overnight before long term storage at −80°C prior to RNA extraction and gene expression analysis.

### RNA extraction

The collected tissue samples were thawed at room temperature and total RNA was extracted using chloroform and Trizol method followed by purification with the Qiagen RNeasy kit (Qiagen, Inc., CA, United States) according to the manufacturer’s instructions. DNA was inactivated using DNAse I Amplification Grade (Invitrogen, Carsbad, CA, United States). Tissue samples that yielded less than 10 ng of total RNA were omitted from further analysis because of insufficient RNA quantity, and this included 16 out of the 32 tissue samples processed.

### Library preparation

cDNA libraries were constructed using Ion Ampliseq Transcriptome Human Gene Expression Kit from Thermo Fisher (MA, United States, Cat# A26325) according to manufacturer’s recommended protocol. Briefly, 10 ng of total RNA was reverse transcribed at 42°C for 30 min. After reverse transcription the cDNA was amplified by PCR using Ion Ampliseq Transcriptome Human Gene Expression Core Panel primers that amplified the specific targets (step 1: 99°C for 2 min; step 2: 99°C for 15 s, 60°C for 16 min for 16 cycles; step 3: Hold at 10°C). Next, the primers were partially digested as directed. Following the partial digestion of primers, adapters and bar codes were ligated to the cDNA. The cDNA was then purified using AMPure XP reagent (Beckman Coulter IN, United States, Cat#A63881) and the recommended protocol. The purified cDNA libraries were then amplified by PCR using 1X Library Amp Mix and 2 uL of 25X Library Amp Primers with the conditions as follows: Step 1: 98°C for 2 min; Step 2: 98°C for 15 s, 64°C for 1 min; steps 2–3 for 5 cycles and then hold at 10°C. The amplified cDNA libraries were purified using Nucleic Acid binding beads, binding buffers and processed on Agilent 2,100 Bioanalyzer (Agilent CA, United States) to determine the yield and size distribution of each library.

### Bioanalyzer library quality control

The quality of each final library was assessed using the Agilent® dsDNA High Sensitivity Kit (Agilent CA, United States, CAT#5067–4,626). All samples had very low RIN numbers (range: 1–3), but they did not differ between the ultrasound and sham groups, so we further investigated sample quality by examining the DNA library directly. A typical Ampliseq Trancriptome library is shown in Supplementary Figure S1. The molar concentration is determined and lower percentages of DNA (50–160 bp range) indicate a higher quality library. Those with less than 50% of the library in this region were deemed acceptable for templating and sequencing. 13 out of the 16 samples were deemed acceptable after the bioanalyzer quality control and were templated and sequenced.

### Templating, enrichment, and sequencing

Approximately 50 pM of pooled barcoded libraries were used for templating using Thermo Fisher Ion 550 Kit-Chef (ThermoFisher, MA, United States, Cat.# A34541) and the manufacturer’s recommended protocol. Briefly, 50 pM of pooled libraries were combined and 25 ul of each sample was loaded onto the Ion Chef. Next, all reagents for the Ion 550 Chef Kit were loaded onto the Ion Chef (ThermoFisher, MA, United States) and the run was performed. The Ion Chef templated, enriched, and loaded the sample onto a 550 chip. After 15 h the Chef paused, and QC was performed on the unenriched samples. The beads were then isolated, and quality assessment was performed on the Qubit instrument (ThermoFisher, MA, United States) to determine the percentage of beads that were polyclonal. All samples at this point passed QC. After polyclonal assessment the Ion Chef resumed running and loaded the samples onto a 550 chip. The loaded chip was then placed into an Ion S5 sequencer, and the run was started using an Ion torrent Ampliseq transcriptome run plan that was configured based on type of library, species, number of run flows required, type of plug-in required, adapter-trimming as well as other parameters specific to the Ampliseq transcriptome run.

### Alignment and data analysis

After completion of the run, the raw sequence files (fastq) were aligned to the human transcriptome (hg19) reference sequences by the StrandNGS (CA, United States) software using the default parameters. The genes and transcript annotations that were used were retrieved from the Ensembl data base. Aligned BAM files were used for further analysis. Quality control was assessed by the Strand NGS program, which determined the pre-and post-alignment quality of the reads for each sample. The aligned reads were then filtered based on alignment score (≥90), match count (≤1), number of N’s allowed in reads (≤0). Aligned reads that failed these vendor quality control guidelines were removed. Only 7 of the 13 samples sequenced showed aligned sequence greater than 60% (vendor quality control guideline). After this filtering, the aligned reads were normalized and quantified using the DEseq algorithm by the StrandNGS program. Next statistical analysis was performed using the Benjamini Hochberg false discovery rate and Moderated T-test to determine significant differentially expressed genes (*p* < 0.05) between ultrasound-treated and sham-treated groups. After significant DEGs were identified, significant fold change between ultrasound treated subjects and sham treated subjects was determined and significant genes with a fold change >4 were clustered. Additionally, genes that had a significant fold change of 2 or higher were listed. Gene Set Enrichment Analysis (GSEA) was performed using the StrandNGS software and Broad Institute databases.

### Data availability

Raw and processed data have been deposited in GEO (accession number GSE222503).

## Results

### Global gene expression analysis

**Figure 2:**
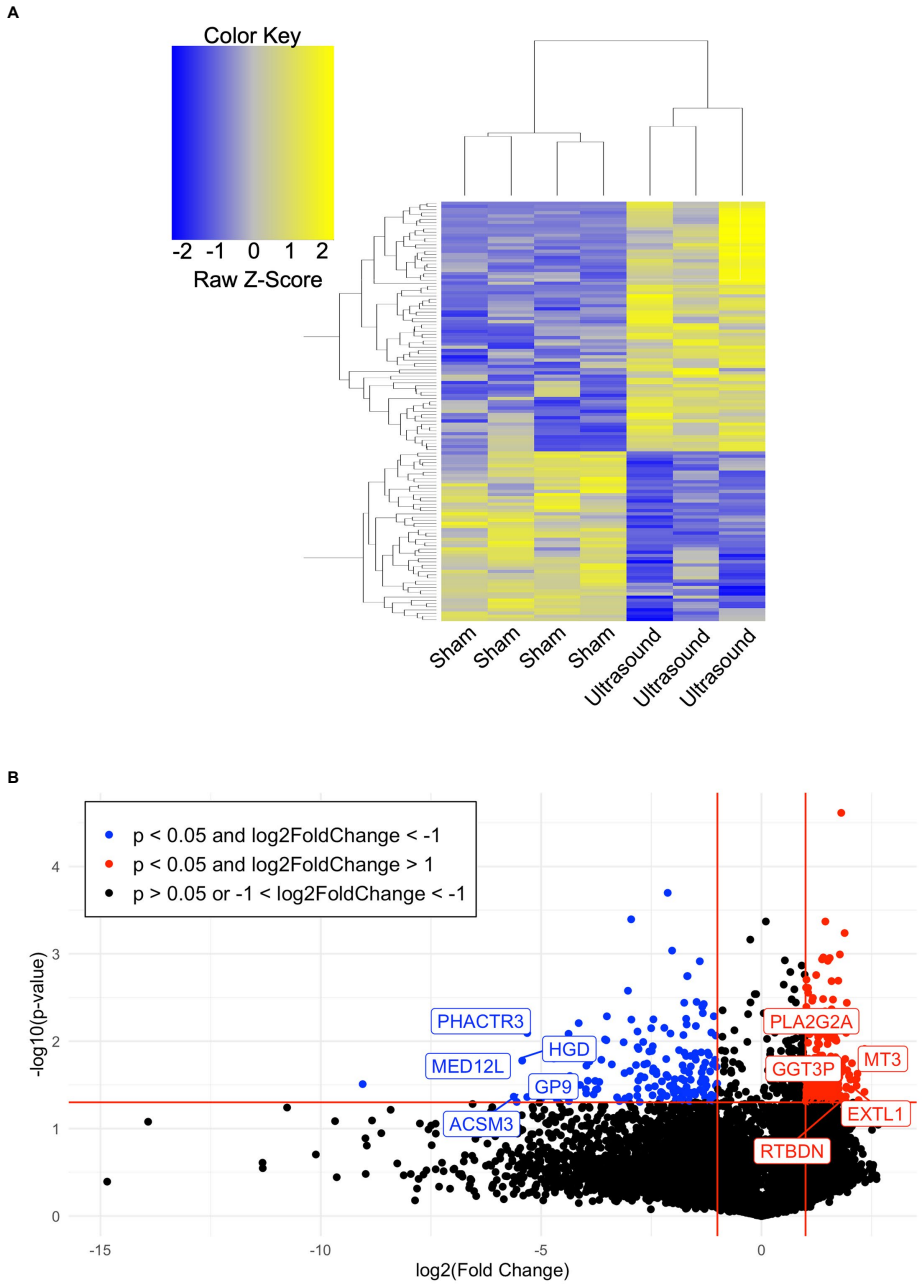
**(A)** Hierarchical clustering of DEGs (*p* < 0.05, absolute value of fold change >4) when comparing the ultrasound to sham group. **(B)** Volcano plot of value of p vs. fold change (FC) of gene expression in ultrasound compared to sham group. Lines indicate FC greater than 2 and *p* < 0.05 using moderated T-test with Benjamini Hochberg FDR multiple correction test.

A total of 7 human venous leg ulcer samples were analyzed (*n* = 3 ultrasound; *n* = 4 sham) for significantly different gene expression when comparing ultrasound-treated patients to sham-treated subjects. 477 significantly differentially expressed genes (DEGs) were identified with a fold change greater than 2 and *p* < 0.05 (Figure 2, Supplementary Tables S1, S2). Hierarchical clustering of DEGs showed that the ultrasound-and sham-treated subjects clustered separately. We have also displayed the top 20 upregulated and downregulated protein encoding DEGs (Table 2). Many of these genes are involved in regulation of metabolism (PLA2G2A, MT3, GGT3P, RTBDN, TM4SF5, PGM3, AWAT1, CYP21A2, SLC8B1, HGD, ACSM3, AK8, PISD, PLB1), cell proliferation (CA9, TM4SF5, RORA, USP51, CGREF1, PHACTR3, CDC25A, UBE2V1), and immune cell signaling (GGT3P, IFIT1B, GPA33, RUFY4, P2RY12, CLEC1B).

**Table 2:** Top 20 upregulated and downregulated protein encoding DEGs (*p* < 0.05) in ultrasound-treated vs. sham-treated wounds.

Upregulated DEGS	*p*-value	Fold change	Function of encoded protein	References
PLA2G2A	0.031	16.623	Phospholipase A2 family (PLA2) which participates in the regulation of the phospholipid metabolism in biomembranes	NCBI
MT3	0.041	12.405	Member of the metallothionein family of genes and plays an important role in zinc and copper homeostasis, and is induced under hypoxic conditions	NCBI, Ref. (21)
GGT3P	0.039	12.222	Involved in peptide metabolic process, response to estradiol, and response to tumor necrosis factor	NCBI
EXTL1	0.043	11.021	Involved in chain elongation of heparin sulfate and possibly heparan. Promotes CCR7-mediated dendritic cell migration to retrain infection and autoimmunity	NCBI, Ref. (22)
RTBDN	0.022	11.019	May play a role in binding retinoids and other carotenoids as it shares homology with riboflavin binding proteins	NCBI
CA9	0.043	10.463	CAs are a large family of zinc metalloenzymes that catalyze the reversible hydration of carbon dioxide. May be involved in cell proliferation and transformation. Regulated by hypoxia.	NCBI, Ref. (23)
TM4SF5	0.022	10.333	Cell surface glycoprotein that plays a role in the regulation of cell development, activation, growth and motility. Modulates hepatocyte and macrophage crosstalk to reprogram the inflammatory environment.	NCBI, Ref. (24)
RORA	0.042	9.297	Member of NR1 subfamily of nuclear hormone receptors and shown to interact with NIM23-2, which is involved in organogenesis and differentiation. Modulates M2 macrophage polarization.	NCBI, Ref. (25)
PGM3	0.014	8.882	Member of the phosphohexose mutase family that may play a role in resistance to diabetic nephropathy and neuropathy	NCBI, Ref. (26)
AWAT1	0.029	8.537	Plays a central role in lipid metabolism in skin and is predominantly expressed in the sebaceous gland of the skin	NCBI
CYP21A2	0.022	8.284	P450 superfamily of enzymes which catalyze reactions involved in drug metabolism and synthesis of cholesterol, steroids, and other lipids	NCBI
KIF5A	0.003	7.245	Member of the kinesin family of proteins that function as a microtubule motor in intracellular organelle transport	NCBI
CEP290	0.003	7.101	Encodes a centrosomal protein	NCBI
SIM2	0.027	6.994	Master regulator of neurogenesis	NCBI
USP51	0.028	6.979	Involved in regulation of cell cycle process and enables chromatin binding activity and histone binding activity	NCBI
CGREF1	0.035	6.793	Predicted to enable calcium ion binding activity and predicted to be involved in negative regulation of cell proliferation	NCBI
OR2T7	0.012	6.645	Olfactory receptor protein	NCBI
TMEM191C	0.015	5.892	Predicted to be an integral component of the cell membrane	NCBI
RALA	0.018	5.735	Protein in the Ras family of proteins which mediate the transmembrane signaling initiated by the occupancy of certain cell surface receptors	NCBI
SLC8B1	0.004	5.608	Belongs to family of potassium-dependent sodium/calcium exchangers that maintain cellular calcium homeostasis	NCBI
Downregulated DEGs	*p*-value	Fold Change	Function of Encoded Protein	References
PHACTR3	0.003	−16.181	Member of the phosphatase and actin regulator protein family and associated with the nuclear scaffold in proliferating cells	NCBI
HGD	0.004	−13.439	Enzyme homogentisate 1,2 dioxygenase and is involved in the catabolism of the amino acids tyrosine and phenylalanine	NCBI
MED12L	0.006	−10.216	Part of the Mediator complex, which is involved in transcriptional coactivation of nearly all RNA polymerase II-dependent genes	NCBI
GP9	0.026	−10.111	A small membrane glycoprotein found on the surface of human platelets and functions as a receptor for von Willebrand factor	NCBI
ACSM3	0.002	−8.454	Predicted to be involved in acyl-CoA metabolic process and fatty acid biosynthetic process	NCBI
AK8	0.001	−7.841	Enables AMP binding activity; involved in nucleoside diphosphate phosphorylation and nucleoside triphosphate biosynthetic process	NCBI
PISD	0.01	−7.607	Catalyzes the conversion of phosphatidylserine to phosphatidylethanolamine in the inner mitochondrial membrane and is active in phospholipid metabolism	NCBI
MID1	0.003	−7.604	Member of the tripartite motif (TRIM) family and likely involved in the formation of multiprotein structures acting as anchor points to microtubules	NCBI, Ref. (27)
SIX2	0.011	−7.586	Regulation of GDNF expression	Ref. (28)
CACNA1I	0.038	−7.511	Member of a subfamily of calcium channels referred to as is a low voltage-activated, T-type, calcium channel and may be involved in calcium signaling in neurons	NCBI
IFIT1B	0.007	−7.433	Predicted to act upstream of or within cellular response to interferon-alpha; cellular response to interferon-beta; and response to bacterium	NCBI
GPA33	0.007	−6.567	Expressed by mature regulatory T cells	Ref. (29)
PLB1	0.012	−6.356	Membrane-associated phospholipase that displays lysophospholipase and phospholipase A2 activities through removal of sn-1 and sn-2 fatty acids of glycerophospholipids	NCBI
KCNQ5	0.007	−6.195	Member of the KCNQ potassium channel gene family that is differentially expressed in subregions of the brain and in skeletal muscle. Currents expressed from this protein have voltage dependences and inhibitor sensitivities in common with M-currents	NCBI
CDC25A	0.015	−5.805	Activates the cyclin-dependent kinase CDC2 by removing two phosphate groups and is required for progression from G1 to the S phase of the cell cycle	NCBI
APOBEC3H	0.007	−5.78	RNA-binding cytidine deaminase that has antiretroviral activity by generating lethal hypermutations in viral genomes and are associated with increased resistance to human immunodeficiency virus type 1 infection	NCBI
RUFY4	0.007	−5.539	Involved in autophagosome assembly; cellular response to interleukin 4; and positive regulation of macroautophagy	NCBI
P2RY12	0.04	−5.419	Involved in platelet aggregation and is a potential target for the treatment of thromboembolisms	NCBI
CLEC1B	0.023	−5.248	Calcium-dependent (C-type) lectin-like receptors that interacts with major histocompatibility complex class I molecules and either inhibit or activate cytotoxicity and cytokine secretion	NCBI
UBE2V1	0.005	−4.986	Involved in the control of differentiation by altering cell cycle behavior	NCBI

**Figure 3:**
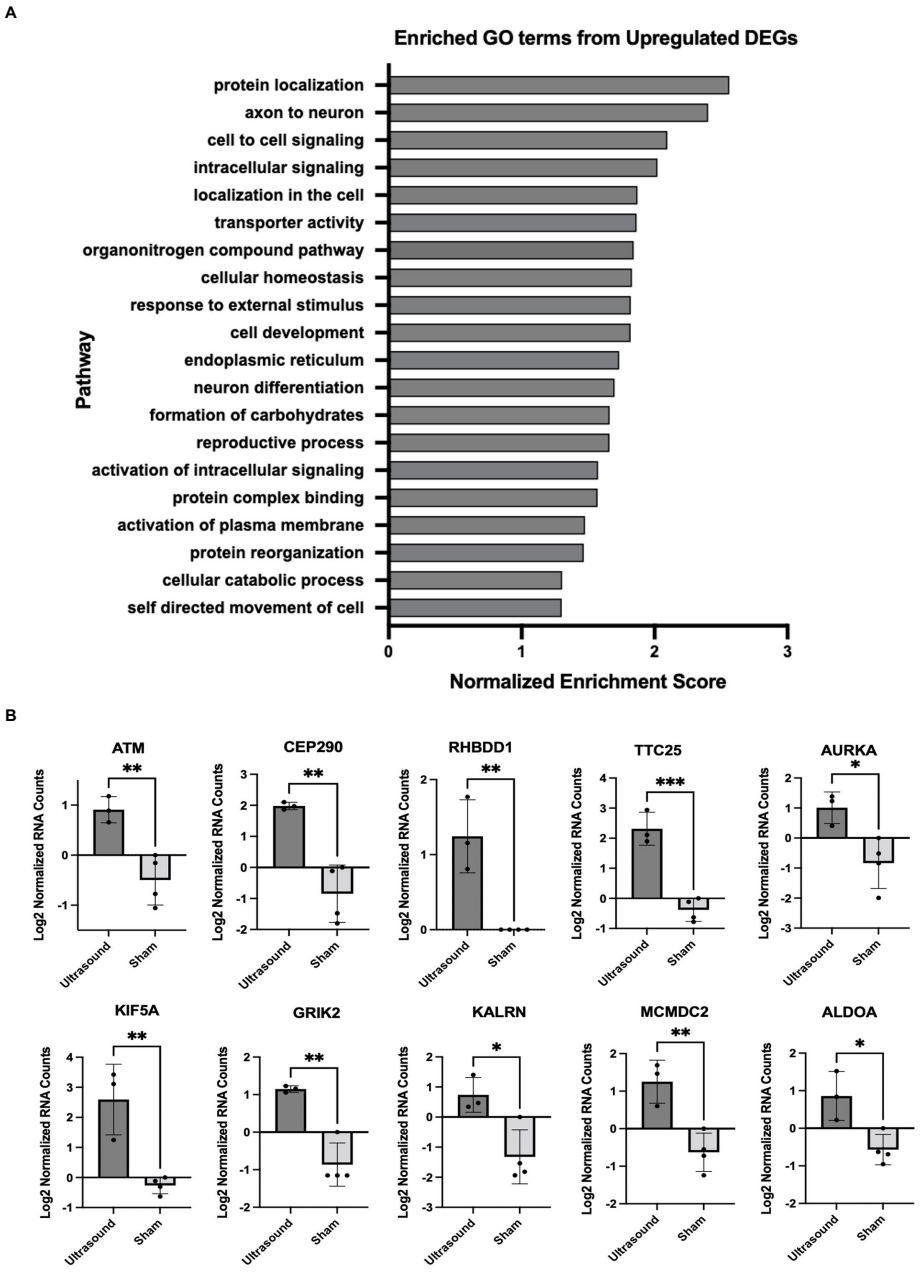
**(A)** Enriched GO terms identified from significantly upregulated DEGs (*p* < 0.05). **(B)** DEGs that contributed the most to the enriched gene sets; *p < 0.05; **p < 0.01, ***p < 0.001.

**Figure 4:**
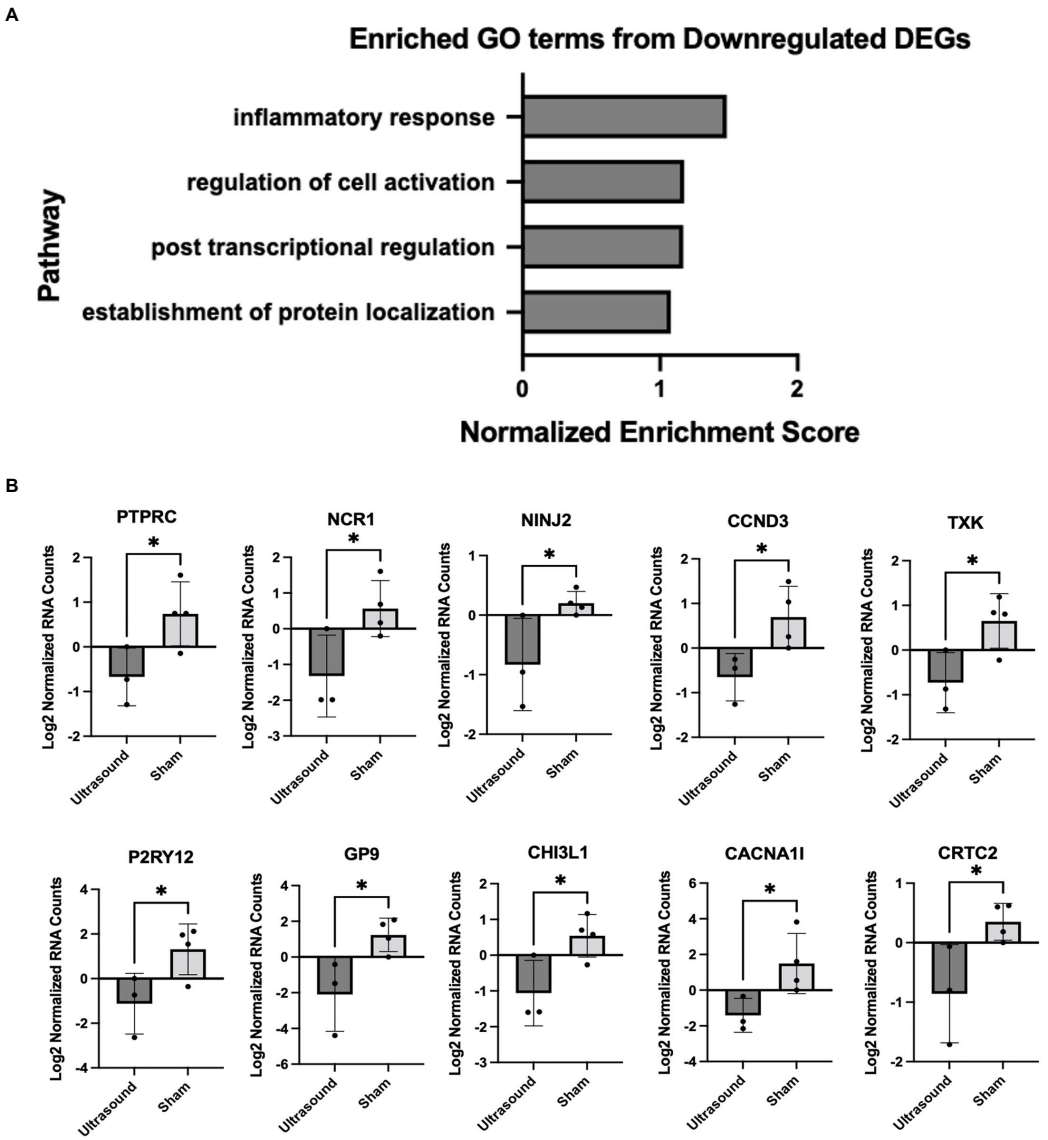
**(A)** Enriched gene sets identified from significantly downregulated DEGs (p < 0.05). **(B)** DEGs that contributed the most to the enriched gene sets; *p < 0.05.

We employed gene set enrichment analysis to determine any significantly enriched sets of genes with similarly grouped functions, using gene sets listed on the Broad Institute database. 20 significantly enriched gene sets from upregulated DEGs were identified (Figure 3A). Ten DEGs that most contributed to the enriched gene sets are shown in Figure 3B. Four significantly enriched gene sets from downregulated DEGs were identified (Figure 4A) with 10 DEGs that most contributed to the enriched gene sets (Figure 4B). Of note, the most enriched gene set from downregulated DEGs was the inflammatory response gene set, which includes 761 hallmark genes.

## Discussion

Gene set enrichment analyses of human chronic venous leg wound tissue showed significant differences in participants treated with LFLI US compared to participants treated with a sham device. These results are important because they provide the foundation for future studies to investigate possible mechanisms of action behind the beneficial effects of ultrasound. In particular, the inflammatory response gene set was significantly downregulated in the ultrasound-treated patients compared to sham-treated patients as early as 1 week after treatment. These results suggest that a decrease in inflammation is a possible biological mechanism of ultrasound-mediated chronic venous leg ulcer wound healing, although this hypothesis will need to be rigorously tested in follow-up studies. These data contribute to the understanding of how therapeutic ultrasound modulates cells in human chronic wounds and are particularly essential in the absence of adequate animal models for human VLUs. With additional understanding of the biological mechanism of therapeutic ultrasound on cells, ultrasound parameters can be tailored to maximize chronic wound healing, ultimately leading to an increase in the quality of life for patients and a decrease in wound care associated costs.

Our findings that LFLI US significantly downregulates the inflammatory response gene set corroborates the findings of Yang et al., who found low-intensity ultrasound to globally activate anti-inflammatory genes revealed through Ingenuity Pathway Analysis (IPA) on *in-vitro* experimental data found in a widely available microRNA database (30). Furthermore, Hundt et al. reported a significant downregulation in genes associated with inflammation after high-intensity (total acoustical power of 120 W) ultrasound treatment applied directly to muscle tissue in mice (31). The downregulation of inflammation would be expected to be beneficial for chronic wound healing as sustained inflammation has been shown to be a major impediment of healing in chronic wounds (32, 33) and decreasing inflammation has been associated with improved healing outcomes in diabetic foot ulcers (17, 34). Modulation of inflammation has been suggested to be a primary mediator of ultrasound-stimulated bone fracture healing (12). Additionally, the top protein encoding genes (Table 2) can be used to motivate future studies in understanding pathway specific mechanisms. For example, many of the upregulated genes in ultrasound-treated wounds were related to metabolism, which has been recently linked to regulation of wound healing in animal models of diabetic wounds (35). Some of these genes are also related to inflammation *via* metabolic regulation. For example, the top upregulated gene, PLA2G2A, is a member of the PLA2 phosolipase family of proteins that regulate synthesis of bioactive lipids that regulate inflammation in wound healing (36). The second most upregulated gene, MT3, encodes metallothionein 3, which is critical for inhibiting the pro-inflammatory phenotype and promoting the “pro-healing” phenotype of macrophages (21). In considering the literature and our findings, we suggest that the possible biological mechanisms of ultrasound-assisted healing may include regulation of inflammation. Additional studies will need to be conducted to determine specific pathways responsible for these effects.

Although our findings identified several statistically significant genes and pathways that were affected by LFLI US, this study should be considered preliminary. While the study started with the recruitment of 48 venous ulcer patients, due to patient loss to follow up and poor tissue quality, the final analysis included samples from only 3 ultrasound-treated and 4 sham-treated subjects, thus limiting the statistical power of the study. Moreover, 0 of the 3 ultrasound-treated subjects and 2 of the 4 sham-treated subjects healed during the study, so it is possible that healing outcome could have influenced the results. However, this risk may be partially reduced by the fact that the samples were collected at 1 week after initial treatment and several weeks away from healing or the end of the study. Additionally, differences in subject demographics could have influenced the results, and the low number of replicates prevented statistical analysis of each factor between groups. Much larger studies are required to definitively conclude that the observed effects result from ultrasound treatment and not some other factor including healing outcome. Technical limitations include that this study analyzed whole tissue, which limits the understanding of individual cell types that contribute to the observed differences, and that the selected platform (Ampliseq) only analyzes protein-encoding genes as opposed to the whole transcriptome. Hence, future work exploring cell specific mechanisms should utilize single cell analysis instead of whole tissue and using whole transcriptomic sequencing if possible. Finally, we only analyzed tissue collected at a single time point (1 week after initial treatment), and other changes would be expected to occur over time. These limitations notwithstanding, these results show that low-frequency, low-intensity therapeutic ultrasound significantly affects human chronic wounds on the cellular level and these results can be used to further develop therapeutic ultrasound and maximize the potential for healing.

## Conclusion

Ultrasound treatment influenced cellular processes in human chronic VLUs as early as 1 week after treatment. Wound tissue samples from subjects treated with ultrasound exhibited increased cell–cell signaling and decreased inflammatory response gene sets. These results provide a foundation for future studies examining these potential mechanisms in depth.

## Data availability statement

The datasets presented in this study can be found in online repositories. The names of the repository/repositories and accession number(s) can be found in the article/Supplementary material.

## Ethics statement

The studies involving human participants were reviewed and approved by Drexel University Institutional Review Board. The patients/participants provided their written informed consent to participate in this study.

## Author contributions

OB, KS, PL, MW, RD-G, and MN designed the study. OB and ZA conducted the experiments and analyzed the data. OB, ZA, and KS wrote the manuscript. All authors contributed to the article and approved the submitted version.

## Funding

This study was supported by NIH F31 AR074847 and NIH R01 NR015995. The content of this study is solely the responsibility of the authors and does not necessarily represent the official views of the National Institutes of Health.

## Conflict of interest

PL declares pending patent applications related to the ultrasound technology described in this work.

The remaining authors declare that the research was conducted in the absence of any commercial or financial relationships that could be construed as a potential conflict of interest.

## Publisher’s note

All claims expressed in this article are solely those of the authors and do not necessarily represent those of their affiliated organizations, or those of the publisher, the editors and the reviewers. Any product that may be evaluated in this article, or claim that may be made by its manufacturer, is not guaranteed or endorsed by the publisher.
