# Supplementary material for: Gene expression changes in therapeutic ultrasound-treated venous leg ulcers
Source: Front Med (Lausanne). 2023 Mar 30;10:1144182. doi: 10.3389/fmed.2023.1144182 (PMC10098114; doi:10.3389/fmed.2023.1144182)
Supplement: Supplementary file 1 [file Data_Sheet_1.PDF]

Supplemental information

Gene Expression Changes in Therapeutic Ultrasound-Treated Human Chronic Wound Tissue

Olivia Boerman et al.

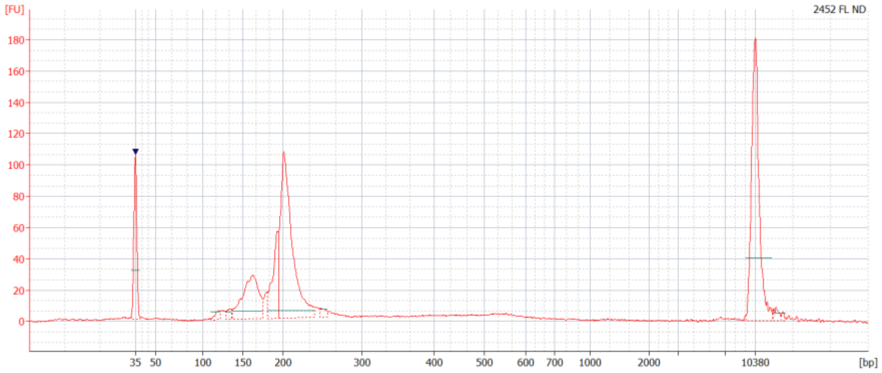

Supplemental Figure 1. Graph displaying desired transcriptome library distribution for samples identified as optimal quality additional processing.

Supplemental Table 1. Table of all identified significantly upregulated genes in ultrasound group compared to sham group.

Gene Symbol	P-value	Fold Change
RP11-383C5.3	0.009	16.725
PLA2G2A	0.031	16.623
MT3	0.041	12.405
GGT3P	0.039	12.222
HIST2H2BD	0.019	11.510
EXTL1	0.043	11.021
RTBDN	0.022	11.019
KRT8P46	0.026	11.013
CA9	0.043	10.463
TM4SF5	0.022	10.333
RORA	0.042	9.297
PGM3	0.014	8.882
AWAT1	0.029	8.537
CYP21A2	0.022	8.284
RP1-274L7.1	0.019	7.889
AC080008.1	0.020	7.795
HNRNPA3P2	0.046	7.454

KIF5A	0.003	7.245
RP11-343H19.2	0.017	7.212
CEP290	0.003	7.101
SIM2	0.027	6.994
CTD-2587H24.5	0.046	6.989
USP51	0.028	6.979
RP11-126F18.2	0.006	6.907
RP4-622L5.7	0.041	6.877
CGREF1	0.035	6.793
OR2T7	0.012	6.645
TTC25	0.000	6.489
CTD-2313N18.5	0.005	6.200
AP001476.2	0.039	5.969
TMEM191C	0.015	5.892
PTPLA	0.013	5.852
RALA	0.018	5.735
LRRC46	0.000	5.678
SLC8B1	0.004	5.608
RP11-159J3.1	0.034	5.529
RP11-302I18.1	0.005	5.484
GPR39	0.001	5.398
C1QTNF2	0.005	5.360
ABCA9	0.004	5.307
RP11-118B18.2	0.020	5.287
CORIN	0.028	5.180
NMNAT2	0.005	5.153
BPIFB1	0.001	5.090
RP11-416I2.1	0.025	5.090
LEMD1	0.022	5.077
VSIG8	0.024	4.972
PSORS1C3	0.044	4.934
ZNF878	0.038	4.888
RP11-461O7.1	0.004	4.822
WDR93	0.024	4.796
RP11-362F19.2	0.007	4.758
RD3	0.030	4.732
RP11-282O18.3	0.032	4.647
AGBL4	0.007	4.640
ELAVL2	0.012	4.619
SOGA3	0.026	4.609
GTF2IRD2P1	0.023	4.606
C4BPB	0.042	4.595
CTD-2293H3.1	0.007	4.531
FRG2C	0.019	4.502
CDK18	0.045	4.469
RP3-337H4.8	0.006	4.415

RP11-1250I15.3	0.036	4.399
RP11-609N14.1	0.029	4.341
ZNF845	0.040	4.280
PPP4R4	0.005	4.246
CMP21-97G8.2	0.038	4.229
ZNF471	0.002	4.227
AC093838.4	0.043	4.218
KALRN	0.014	4.172
IL17B	0.032	4.126
EDNRA	0.047	4.083
RP3-499B10.3	0.017	4.082
NEURL3	0.018	4.045
GRIK2	0.001	4.030
NUDT1	0.014	4.019
TMF1	0.026	4.001
AC113167.1	0.016	3.995
RP11-386I23.1	0.007	3.989
RP13-578N3.3	0.048	3.989
SLC13A3	0.036	3.982
CTD-2542L18.1	0.019	3.978
RP11-564A8.4	0.049	3.963
COL8A2	0.013	3.951
CCL16	0.019	3.949
AC017076.5	0.046	3.949
ITGB1BP2	0.011	3.937
RP11-53B2.2	0.033	3.927
PTOV1-AS1	0.020	3.915
MICALL2	0.012	3.909
AC020922.1	0.006	3.892
AC009499.1	0.013	3.870
ANGPTL6	0.044	3.869
PRSS45	0.001	3.801
CTC-573M9.1	0.038	3.794
RP11-394I13.2	0.006	3.739
PTPN5	0.029	3.705
MCMDC2	0.004	3.685
RP11-169K16.9	0.001	3.683
PPP1R3F	0.007	3.673
AP000662.4	0.016	3.659
GSTO2	0.045	3.659
RP11-429J17.2	0.039	3.649
DHRS12	0.006	3.646
WNT9A	0.037	3.643
AURKA	0.017	3.595
CYB5RL	0.027	3.585
PLA2G4C	0.001	3.575

RP11-455G16.1	0.035	3.567
ITPKA	0.047	3.550
FRY-AS1	0.008	3.549
GLULP4	0.019	3.547
TIMM8A	0.014	3.531
PDILT	0.007	3.531
THUMPD1	0.046	3.499
FAM131B	0.006	3.494
SHISA2	0.028	3.456
ALMS1-IT1	0.023	3.449
RP11-798M19.6	0.016	3.434
SH3GL1	0.014	3.407
STX1A	0.043	3.394
FKBPL	0.039	3.387
SBSPON	0.030	3.372
ZC3H12B	0.019	3.362
RP11-893F2.5	0.049	3.348
RP1-212P9.2	0.000	3.321
AC005086.2	0.017	3.320
SCN2B	0.003	3.289
RP11-102M11.2	0.025	3.269
RP11-227G15.9	0.018	3.258
AC002310.13	0.047	3.254
RP11-61A14.4	0.021	3.250
ZNF232	0.048	3.246
TNNI1	0.028	3.237
LLPH	0.028	3.228
RP11-430N14.4	0.004	3.208
FAM71F2	0.044	3.182
NLRP8	0.013	3.147
IL5RA	0.021	3.145
RPS15P5	0.022	3.132
ABO	0.001	3.126
BTG3	0.048	3.120
CTC-550B14.7	0.002	3.108
ADCY4	0.046	3.085
FABP7P2	0.026	3.078
AC018865.8	0.027	3.060
RP11-492E3.2	0.028	3.057
RPL7P4	0.006	3.051
RP11-545A16.1	0.006	3.050
CA11	0.033	3.048
PTGIR	0.044	3.046
RP5-1166F10.1	0.011	3.042
CYP17A1	0.040	3.036
ZNF606	0.029	3.020

SFTA2	0.038	3.017
MCHR2	0.001	3.017
RP6-206I17.1	0.017	3.009
WBP2NL	0.026	3.001
HS3ST3B1	0.041	3.000
SMAD9	0.046	2.983
CADPS	0.047	2.960
AICDA	0.024	2.921
GALNT16	0.043	2.919
RNF212	0.020	2.901
CICP27	0.041	2.884
RP11-723D22.3	0.029	2.883
RP11-283I3.6	0.019	2.875
FAM173A	0.042	2.866
RP11-342C24.8	0.011	2.849
RP11-382D8.5	0.016	2.846
C6ORF165	0.020	2.843
OR2H1	0.046	2.839
ZNF720	0.036	2.798
KB-1471A8.1	0.019	2.797
ADAM29	0.021	2.788
RP11-445O3.3	0.023	2.785
GS1-259H13.2	0.008	2.777
LINC00996	0.029	2.769
PEX5L	0.023	2.752
RN7SL275P	0.031	2.747
RP11-475A13.1	0.049	2.740
KLHDC1	0.028	2.735
AC010970.2	0.007	2.719
XXbac-B444P24.8	0.024	2.708
RP11-1094M14.11	0.042	2.702
WIPF3	0.045	2.702
RP11-814P5.1	0.008	2.692
ALDOA	0.012	2.691
CA14	0.043	2.669
FAM157A	0.034	2.657
INTU	0.015	2.654
C9orf117	0.035	2.650
ATM	0.005	2.648
RP11-205M3.3	0.043	2.639
FBXL12	0.042	2.633
TRAF5	0.019	2.629
C1orf220	0.027	2.621
C1ORF220	0.027	2.621
LONRF2	0.041	2.601

TMEM132B	0.047	2.601
DEF8	0.033	2.588
DPH3P1	0.009	2.579
RN7SL59P	0.001	2.574
FAM166A	0.035	2.566
RP11-169D4.1	0.021	2.532
AL359878.1	0.018	2.521
AC018506.1	0.019	2.519
ING1	0.016	2.508
EXOSC6	0.026	2.501
RP11-356C4.3	0.017	2.496
ZSCAN10	0.004	2.496
RP11-521B24.3	0.048	2.493
CTD-2369P2.2	0.012	2.485
CORO2B	0.032	2.475
TCEANC2	0.027	2.474
TAPBP	0.048	2.470
AHNAK	0.030	2.469
CCDC113	0.032	2.465
KRTCAP2	0.005	2.451
CDH17	0.045	2.434
AGXT	0.034	2.428
LL22NC03-63E9.3	0.036	2.415
RUNX1	0.012	2.410
TTC16	0.030	2.395
CEP112	0.018	2.390
ZNF90P1	0.034	2.390
RP3-368A4.5	0.041	2.388
C19orf18	0.016	2.377
RP5-961K14.2	0.023	2.370
RHBDD1	0.002	2.369
GGT6	0.046	2.364
AP4M1	0.042	2.364
FLJ27365	0.017	2.355
PARPBP	0.044	2.355
DDX50P1	0.032	2.354
AC003102.3	0.047	2.341
BOC	0.042	2.340
ZNF814	0.025	2.339
SPTY2D1-AS1	0.016	2.332
AAED1	0.002	2.332
RP11-579E24.1	0.021	2.331
LNP1	0.042	2.320
AC074117.10	0.034	2.313
RP5-930J4.4	0.013	2.311
GUSBP2	0.049	2.299

AC092933.4	0.040	2.298
MXRA7	0.033	2.295
SPEF2	0.024	2.287
VPS37A	0.017	2.282
DAW1	0.014	2.277
CTD-2328D6.1	0.004	2.271
CENPE	0.016	2.266
TM6SF2	0.014	2.250
DZANK1	0.035	2.246
RC3H1-IT1	0.003	2.229
RP4-591N18.2	0.036	2.227
AL161915.1	0.027	2.224
HFE	0.041	2.205
N4BP2	0.037	2.204
C1orf85	0.050	2.203
RP4-535B20.4	0.006	2.190
LRRC34	0.022	2.189
RP11-273G15.2	0.016	2.182
RBBP8	0.041	2.179
RN7SL669P	0.030	2.177
NME9	0.044	2.176
HLA-L	0.018	2.164
CENPQ	0.040	2.157
NPAP1P2	0.030	2.149
CTC-338M12.6	0.012	2.144
PPCS	0.047	2.143
PLA2G12A	0.040	2.132
C12orf60	0.044	2.132
RP11-219G17.4	0.037	2.124
AL135901.1	0.002	2.119
CD109	0.040	2.118
TBCCD1	0.008	2.116
NAB2	0.030	2.110
ITGB8	0.023	2.106
USP46	0.025	2.105
RNF144A	0.025	2.105
AC006129.2	0.024	2.092
PLEKHH3	0.049	2.085
POLK	0.037	2.082
RP11-1020A11.2	0.049	2.073
DDX19A	0.024	2.070
NAGPA	0.026	2.063
RP11-84A19.4	0.049	2.062
ZC3H12C	0.035	2.061
FASTKD5	0.040	2.056
SCNN1D	0.041	2.052

RP11-547D24.1	0.032	2.047
RP11-181C3.1	0.040	2.047
FADS1	0.033	2.045
SLC30A5	0.001	2.040
CAMK2A	0.035	2.039
UTP11L	0.003	2.035
ZSCAN12	0.005	2.033
COMMD2	0.030	2.032
SLC22A5	0.002	2.024
RP11-466A19.1	0.023	2.013
BHMT	0.013	2.011
BAIAP2-AS1	0.040	2.008

Supplemental Table 2. Table of all identified significantly downregulated genes in ultrasound group compared to sham group.

Gene Symbol	P-value	Fold Change
PHACTR3	0.003	-16.181
HGD	0.004	-13.439
RP11-1415C14.4	0.019	-12.600
ARSE	0.006	-10.844
RP11-255J3.2	0.046	-10.737
MED12L	0.006	-10.216
GP9	0.026	-10.111
SLAIN1	0.016	-9.013
ACSM3	0.002	-8.454
DDX11L10	0.028	-8.077
AK8	0.001	-7.841
PISD	0.010	-7.607
MID1	0.003	-7.604
SIX2	0.011	-7.586
CACNA1I	0.038	-7.511
IFIT1B	0.007	-7.433
RPL23AP7	0.018	-7.035
GPA33	0.007	-6.567
PLB1	0.012	-6.356
KCNQ5	0.007	-6.195
CCDC74B	0.015	-6.052
CLCN4	0.010	-5.822
DDX11L2	0.050	-5.815
CDC25A	0.015	-5.805
APOBEC3H	0.007	-5.780
RUFY4	0.007	-5.539
P2RY12	0.040	-5.419
CLEC1B	0.023	-5.248

PRRG3	0.045	-5.170
UBE2V1	0.005	-4.986
TUBB1	0.026	-4.854
RBM15	0.032	-4.841
CPNE5	0.047	-4.680
CCNI2	0.003	-4.608
MAP3K7CL	0.021	-4.589
ZDHHC20	0.028	-4.581
C1orf228	0.023	-4.575
LRCH2	0.023	-4.463
PF4	0.010	-4.443
TUBA8	0.011	-4.424
PGR	0.046	-4.366
DDX11L5	0.035	-4.361
C2orf88	0.048	-4.336
SKA3	0.017	-4.307
TCF15	0.007	-4.261
ANKRD46	0.000	-4.201
TMEM155	0.034	-4.198
DPY19L2	0.027	-4.137
ZSCAN16	0.030	-4.101
SWT1	0.003	-4.099
RP11-589F5.4	0.049	-4.080
COL21A1	0.046	-4.043
AVPR1A	0.043	-4.008
MAK	0.037	-3.797
LIMD1-AS1	0.002	-3.784
PRKCQ	0.002	-3.733
NCR1	0.039	-3.700
CD36	0.047	-3.696
USPL1	0.038	-3.595
TMTC1	0.002	-3.583
RAD51C	0.000	-3.577
SYNC	0.044	-3.550
GPR89B	0.003	-3.507
KCNE4	0.049	-3.448
PROC	0.031	-3.439
SUSD3	0.043	-3.408
USP19	0.016	-3.333
SEMG1	0.044	-3.329
KLRB1	0.014	-3.279
WDR37	0.014	-3.249
EPB41	0.024	-3.176
CBX1	0.013	-3.113
CXCR6	0.018	-3.079
HEMGN	0.017	-3.071

LY6G5C	0.026	-3.069
AL390877.1	0.040	-3.061
CHI3L1	0.030	-3.041
CEP85	0.033	-3.032
PELI1	0.033	-2.992
MIF4GD	0.044	-2.981
RILPL1	0.040	-2.960
DHRS13	0.047	-2.956
PPBP	0.013	-2.949
GEMIN2	0.017	-2.933
TRAPPC3L	0.010	-2.897
SPATA7	0.032	-2.886
ZBP1	0.013	-2.793
MPND	0.046	-2.781
CPEB4	0.004	-2.775
RAB20	0.042	-2.768
HDC	0.048	-2.761
GAS2L3	0.034	-2.740
CDKL1	0.017	-2.724
SCFD2	0.010	-2.694
KLHL15	0.022	-2.667
RPS11P5	0.028	-2.666
ANKRD18B	0.022	-2.662
PRIM1	0.036	-2.662
PTPRC	0.036	-2.661
NT5C3A	0.003	-2.635
CD244	0.021	-2.631
C18orf25	0.010	-2.617
KIF15	0.024	-2.612
TXK	0.029	-2.605
FANCB	0.043	-2.600
ZNF22	0.005	-2.560
SELPLG	0.013	-2.560
CCND3	0.031	-2.546
ENKD1	0.049	-2.512
CDKAL1	0.022	-2.512
HNRNPH2	0.027	-2.476
ABHD13	0.017	-2.457
GNG7	0.030	-2.445
RILP	0.015	-2.444
UBAP2	0.010	-2.418
TCF20	0.030	-2.411
DENND4A	0.030	-2.389
L3MBTL3	0.022	-2.388
DCAF6	0.030	-2.379
FBXO33	0.025	-2.378

GPR89A	0.000	-2.344
RP11-341G5.2	0.012	-2.335
IRS2	0.048	-2.322
CRTC2	0.033	-2.314
ZNF837	0.031	-2.307
VSIG10	0.038	-2.302
CD27-AS1	0.044	-2.291
CNNM4	0.026	-2.277
HIST1H2BK	0.004	-2.270
INTS2	0.017	-2.268
THRAP3	0.045	-2.255
ZNF85	0.014	-2.243
C20orf112	0.014	-2.232
CHPT1	0.008	-2.228
R3HDM4	0.020	-2.218
TMEM164	0.022	-2.210
UBALD1	0.009	-2.208
BCL2L11	0.015	-2.202
SETD2	0.050	-2.197
HP1BP3	0.004	-2.187
PIM2	0.000	-2.187
RP11-33B1.1	0.017	-2.183
NECAP1	0.037	-2.170
UBALD2	0.018	-2.167
EIF1B	0.004	-2.126
CASC3	0.007	-2.124
ITK	0.045	-2.116
MSNP1	0.013	-2.115
CCDC28A	0.023	-2.111
SH3BP4	0.029	-2.108
TMEM110-MUSTN1	0.047	-2.094
RFXAP	0.023	-2.092
ZNF516	0.015	-2.090
TMEM110	0.047	-2.085
AC011242.5	0.048	-2.084
SEC14L1	0.025	-2.081
EMC3	0.004	-2.080
GABARAPL2	0.020	-2.069
PMS2P9	0.040	-2.068
RP11-474G23.1	0.006	-2.068
HERPUD2	0.043	-2.059
PNISR	0.038	-2.056
PPP3R1	0.007	-2.055
SURF1	0.012	-2.054
MFSD8	0.030	-2.053

NINJ2	0.038	-2.044
STAU1	0.042	-2.043
MKL1	0.047	-2.038
GYPA	0.036	-2.035
DPH2	0.006	-2.035
MAX	0.025	-2.003
C1orf189	0.048	-2.001
